# AS YOUNG AS YOU FEEL? TRACKING SUBJECTIVE AGE AND PHYSICAL WELL-BEING BEFORE A LUNG CANCER SCAN

**DOI:** 10.1093/geroni/igae098.3266

**Published:** 2024-12-31

**Authors:** Victoria Dunsmore, Shevaun Neupert

**Affiliations:** University of North Carolina, Chapel Hill, North Carolina, United States; North Carolina State University, Raleigh, North Carolina, United States

## Abstract

Research has not yet considered how cancer-related events trigger poor quality of life. Here, researchers examined whether the discrepancy between felt and chronological age was related to physical well-being, and if these fluctuated as patients with lung cancer approached a triggering event – repeated CT scans. 25 patients treated with curative intent treatment for lung cancer were recruited within 6 months of their upcoming CT scan. Patients were predominantly white (80%; Black: 12%), women (96%), between 43 and 78 years old (M = 62.33, SD = 8.10). Baseline surveys collected age, and repeated monthly surveys gathered physical well-being and felt age every 30 days until one’s scan. Following a monthly response rate of 100% (59 of 59 received), researchers calculated proportional discrepancy felt age scores by subtracting chronological age from felt age and dividing by chronological age. Using this score, researchers investigated the monthly relationship between age discrepancy and physical well-being. Results showed that on months when patients felt closer to, or older than, their chronological age they reported increases in physical well-being (γ10 = 1.75, t = 2.13, p =.04). The present model accounted for 33% of the within-person, and 39% of the between-person variance of physical well-being. In contrast to studies in non-clinical populations, results showed that feeling older was associated with better physical well-being in the months prior to a CT scan. Qualitative interviews could identify themes in coping with repeated scans that may mitigate the effects cancer treatment can have on physical well-being.

